# Evaluation of the Quality and Safety of Venous Thromboembolism Prophylaxis Among Gastroenterology Inpatients at a Tertiary Hospital in Australia

**DOI:** 10.1177/00185787231172385

**Published:** 2023-05-20

**Authors:** Nameer van Oosterom, Keshia R. De Guzman, Karl Winckel, Anissa Johnson, Nazanin Falconer

**Affiliations:** 1 University of Queensland, Brisbane, QLD, Australia

**Keywords:** anticoagulants, gastrointestinal disorders, medication safety, drug/medical use evaluation, clinical pathways

## Abstract

**Background and objective**: Hospital acquired venous thromboembolisms (VTEs) are common and preventable. The Queensland Health VTE prophylaxis guidelines, developed in 2018, provide guidance for risk assessment, and prescribing of anticoagulation for prophylaxis and treatment of hospital inpatients. Currently, there are limited recommendations for gastroenterology patients. This study investigated the completion of VTE risk assessments, and the appropriateness of VTE prophylaxis regimens, in accordance with Queensland Health guidelines for gastroenterology patients. The quality and safety of VTE prophylaxis regimens was assessed based on their VTE risk and bleeding risk. **Method**: A retrospective study was conducted by obtaining a random sample of gastroenterology patients admitted to a tertiary Australian hospital, from 1st May 2019 and 1st May 2020, to determine the compliance of VTE risk assessment and thromboprophylaxis prescribing with state-wide VTE guidelines. The quality and safety of thromboprophylaxis was evaluated using the modified Caprini and HASBLED scores, and subsequent thromboprophylaxis-related complications. **Results**: Of the 94 patients reviewed, 68 did not have contraindications to thromboprophylaxis. Of these 68 patients, 32 (47%) had no VTE risk assessment recorded in their clinical records and were not prescribed any thromboprophylaxis during the hospitalization. There was no significant difference between thromboprophylaxis prescribing for patients with low VTE risk, compared to moderate to high VTE risk (*P* = .075). There was a trend for decrease in thromboprophylaxis prescribing as HASBLED bleeding risk score increased, and patients with moderate-high bleed risk were less likely to be prescribed thromboprophylaxis (*P* = .006). There were no thromboprophylaxis related complications identified. **Conclusion:** It is essential that all patients have a clearly documented risk assessment and are prescribed thromboprophylaxis according to best practice guidelines. The prescription of venous thromboembolism prophylaxis should continue to be individualized, with each patient assessed holistically.

## Introduction

Venous thromboembolism (VTE) results in poor patient outcomes such as increased morbidity, mortality, length of stay (LOS); as well as increased costs to the health system.^1^ As VTEs are a leading cause of preventable death in Australia, the Australian Commission on Safety and Quality in Health Care mandates hospitals to implement measures that reduce VTEs.^1,2^ VTE risk assessments for hospitalized patients play a key role in preventing VTE complications, and mortality, by providing thromboprophylaxis guidelines.^3-6^

Local and international guidelines recommend using tools such as the Caprini score to determine VTE risk.^7,8^ The Caprini score is a risk tool for surgical patients that uses 39 risk factors, weighted and summed, to a achieve an overall VTE risk score; higher scores indicate greater VTE risk.^9^ In addition to VTE risk, bleeding risk should also be considered prior to prescribing anticoagulation for thromboprophylaxis. Currently, there are no formalized risk calculators for approximating bleeding risk secondary to thromboprophylaxis. However, the HASBLED score has been developed to estimate bleeding risk for anticoagulation treatment for atrial fibrillation.^10^ Although not specific to prescribing thromboprophylaxis, the HASBLED may have utility in other patient cohorts.^11,12^

If thromboprophylaxis is required according to VTE risk assessment, guidelines can be used to determine the most appropriate regimen. However, various guidelines have different thromboprophylaxis recommendations for low molecular weight heparin (LMWH) and unfractionated heparin (UFH) at varying doses and frequencies.^3-5,8,13^ Compared to UFH, LMWH has a lower risk of heparin induced thrombocytopenia, with equal or greater efficacy.^8,13,14^ As a result, LMWH is often preferred over UFH. However, UFH is often used in patients with renal impairment or high bleeding risk due to hepatic metabolism. It has a shorter half-life, and its actions are reversible with protamine sulfate.^8,13,14^

VTE guidelines often have thromboprophylaxis recommendations for specific patient cohorts. Currently, there are limited recommendations for gastroenterology patients and the Caprini score has not been validated in this cohort. This is problematic due to their high risk of both VTEs and bleeding. With the lack of thromboprophylaxis guidelines in this high-risk cohort, the documentation of VTE risk assessments and the appropriateness of thromboprophylaxis regimens within this clinical setting is unknown, particularly in an Australian setting. This study investigated the completion of VTE risk assessments, and the appropriateness of VTE prophylaxis regimens, in accordance with Queensland Health guidelines for gastroenterology patients. The quality and safety of prescribed thromboprophylaxis regimens was assessed based on their VTE risk and bleeding risk.

## Method

A retrospective observational study of gastroenterology patients who were prescribed VTE thromboprophylaxis in a major tertiary hospital in Queensland, Australia was conducted. A list of all patients admitted under the gastroenterology team between 1^st^ May 2019 and 1^st^ May 2020 was generated, and a random subset of patients were selected. The electronic medical records of these patients were then manually reviewed for inclusion. Patients were excluded if their LOS was <24 hours, if they were not being treated by a gastroenterology specialty, if they were using therapeutic anticoagulation prior to admission, or if there was missing documentation that made it impossible to calculate their VTE risk.

Data collected included patient socio-demographic characteristics such as age, gender, height, and weight; patient admission details, such as principal diagnosis, LOS, and specialty; and clinical characteristics such as concurrent use of antiplatelets and non-steroidal anti-inflammatory drugs, VTE history (ie, past VTE or VTE during the current admission) and laboratory tests (Appendix A). Laboratory test results for glomerular filtration rate (eGFR), platelet count, and hemoglobin (Hb) level were collected both at baseline (at admission) and at discharge. Any documentation of a re-admission to the same hospital within 30 to 60 days was collected. This timeframe was chosen because the risk of developing a VTE is highest in the first 2 months post-discharge.^6^

### Data Analysis

The VTE risk and bleeding risk for each patient, according to applicable risk factors, was calculated using a modified Caprini score, and the HASBLED score (Appendix B).^7,9^ The modified Caprini score was used, instead of the original Caprini score, because all 31 specific variables required for the original Caprini score were not documented. Each patient was allocated a modified Caprini score out of 13; ≤3 indicated low VTE risk, 3 to 4 indicated moderate VTE risk, and ≥5 indicated high VTE risk.^8^ The HASBLED score is only validated for bleeding risk for atrial fibrillation; however, it was used as a surrogate marker of bleeding risk for gastroenterology patients as there is no formalized risk-assessment tool for these patients.^10^ Each patient was allocated a HASBLED score out of 9; higher scores were indicative of higher bleeding risk.^10^ Thromboprophylaxis-related complications were determined by changes in hemoglobin level and platelet count from baseline to discharge. A reduction in platelets of 50%, and a decrease in Hb levels of 20 g/L, were chosen as clinically significant markers for medication harm secondary to thromboprophylaxis.^15,16^

Descriptive statistics were presented for clinical and socio-demographic characteristics; and thromboprophylaxis prescription according to Caprini and HASBLED scores. Chi-squared ( $\chi^{2}$ ) tests of independence were used to compare results for prescribed thromboprophylaxis for low VTE risk versus moderate-high VTE risk, and for low versus moderate-high bleeding risk. Paired *t*-tests were used to determine if the mean change in hemoglobin and platelet level differed significantly between patients that were and were not prescribed thromboprophylaxis. Statistical significance was considered at the *P* < .05 level. All analysis was undertaken using Microsoft Excel and STATA16.

## Results

A random sample of 271 patients were reviewed and 177 were excluded: Eighty-seven due to LOS <24 hours, 81 not under gastroenterology, 6 due to therapeutic anticoagulation prior to admission, and 3 due to missing documentation. This resulted in a final sample of 94 patients that met the final inclusion criteria. Patients had a mean (SD) age of 58.3 (±20) years and a mean (SD) body-mass-index (BMI) of 26.9 (±7.1) kg/m^2^, while 43 (45.7%) were female (Table 1). The LOS was 5.4 (±3.9)days and 72 (77%) of patients had surgery during their admission. The most common presenting complaints were upper gastrointestinal disorders (29%) and gastrointestinal bleeds (27%).

**Table 1.** Clinical and Socio-Demographic Patient Characteristics (n = 94).

	n = 94, Mean (SD)
Socio-demographic characteristic	
Age (years)	58.3 (20.0)
Length of Stay (LOS) (days) (mean ± SD)	5.4 (3.9)
Height (m)	1.69 (0.1)*
Weight (kg)	77.4 (22.2)**
Calculated BMI (kg/m^2^)	26.9 (7.1)
Gender	N (%)
Male	51 (54.3)
Female	43 (45.7)
Clinical characteristics	
Patients who underwent surgery	72 (76.6)
Presenting complaint	
Bleed (PUD, Anemia, Melaena, UGIB, Haematemesis)	25 (26.6)
Cancer (Ampullary, Oesophageal & Neuroendocrine tumors)	4 (4.3)
Colon disorders (Colonic Polyps, Diarrhea, Abdominal pain)	10 (10.6)
Crohn’s disease	7 (7.5)
Infection (Gastroenteritis, Pancreatitis, Candidiasis oesophagitis)	4 (4.3)
Other (Ingestion of foreign bodies, follow-ups, poisonings)	6 (6.4)
Ulcerative Colitis	11 (11.7)
Upper GI disorders (Barrett’s oesophagus, Choledocholithiasis, Cholangitis, Achalasia)	27 (28.7)

*Note.* GI = gastrointestinal, UGIB = Upper gastrointestinal bleed, PUD = peptic ulcer disease.

* One patient did not have height recorded

** Two patient did not have weight recorded

### Overall VTE Risk, Bleeding Risk, and Thromboprophylaxis Prescription

Of the 94 inpatients, 26 (28%) had documented contraindications for prescribed thromboprophylaxis because of active bleeding. Therefore, 68 patients had with no contraindications to thromboprophylaxis. Of these 68 patients, 32 (47%) were not prescribed thromboprophylaxis and did not have documentation of VTE risk assessment recorded. Overall, patients had a high VTE risk with a mean (±SD) modified Caprini score of 5.4 (±1.8), and a lower bleeding risk with a mean (±SD) HASBLED score of 2.5 (±1.3) (Table 2).

**Table 2.** Venous Thromboembolism (VTE) Risk assessment and Bleeding Risk Assessment for Gastroenterology Inpatients (n = 94).

		Mean (SD) 5.4 (1.8)
Applicable risk factor	Weighted score associated with VTE risk factor	N (%)
Overall modified Caprini Score (VTE risk assessment)		
Age 41-60	1	26
Age 61-74 y	2	13
Age >75 y	3	8
Minor surgery	1	73
History of VTE	3	2
Length of stay >3 d	2	38
Body Mass Index >25 kg/m^2^	1	53
History of cancer	2	8
Inflammatory bowel disease	1	24
		Mean (SD) 2.5 (1.3)
Applicable risk factor	Weighted score associated with bleeding risk factor	N (%)
Overall HASBLED score (Bleeding risk assessment)		
Hypertension	1	33
Renal	1	2
Liver	1	10
Stroke	1	5
History of bleeding	1	36
High INR	1	17
Age >65 y	1	38
Medications	1	30
EtOH	1	8

### The Use of VTE Prophylaxis Based on Thrombotic Risk

**Figure 1.**
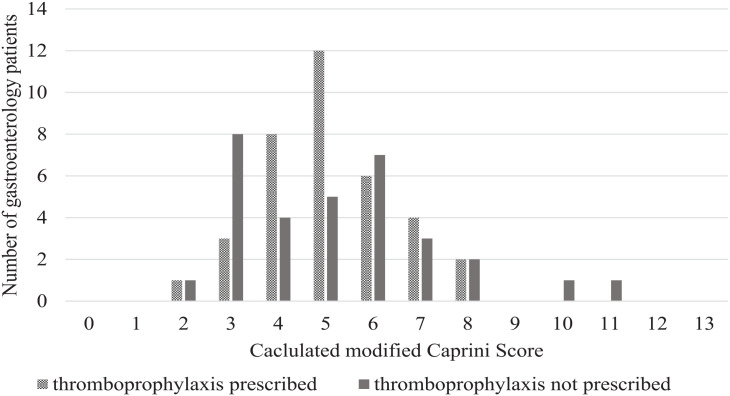
Thromboprophylaxis prescription according to calculated modified Caprini scores for gastroenterology patients with no contraindications to thromboprophylaxis (n = 68).

The use of VTE prophylaxis based on Caprini scores is shown (Figure 1). There were 13 patients with a low VTE risk, of which 4 (31%) were prescribed thromboprophylaxis. There were 55 patients with moderate to high VTE risk and 32 (58%) were prescribed thromboprophylaxis. When comparing the proportion of thromboprophylaxis prescribing between patients with low VTE risk and moderate-high VTE risk, no significant difference between thromboprophylaxis prescribing according to VTE risk was found (*P* = .075) (Table 3).

**Table 3.** Comparison of Thromboprophylaxis Prescribing by VTE Risk and Bleeding Risk for Gastroenterology Inpatients (n = 68).

Thromboprophylaxis prescribed for patients with low or moderate-high VTE risk			
	Low VTE risk	Mod-High VTE risk	Chi-squared result
	Modified Caprini Score = 0 to 3	Modified Caprini Score = 4 to 11	
	(n = 13)	(n = 55)	
	N (%)	N (%)	
Thromboprophylaxis prescribed	4 (31)	32 (58)	$X^{2}$ = 3.17
			*P* = .075
Thromboprophylaxis prescribed for patients with low or moderate-high bleeding risk			
	Low bleeding risk	Mod-High bleeding risk	Chi-squared result
	*HASBLED Score = 0*-*1*	*HASBLED* score* =* score ≥ 2	
	(n = 14)	(n = 54)	
	N (%)	N (%)	
Thromboprophylaxis prescribed	12 (85)	24 (44)	$X^{2}$ =7.60
			*P* = .006

### The Use of VTE Prophylaxis Based on Bleeding Risk

**Figure 2.**
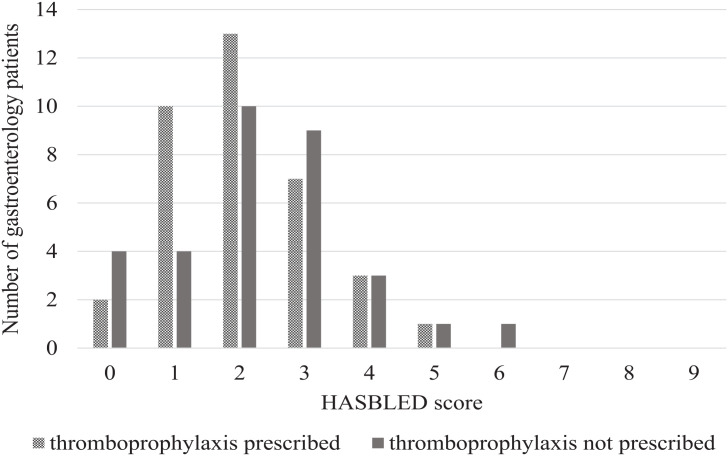
Thromboprophylaxis prescription according to calculated HASBLED scores for gastroenterology patients with no contraindications to thromboprophylaxis (n = 68).

The use of VTE prophylaxis based on HASBLED scores had a trend for decreased thromboprophylaxis prescribing as bleeding risk increased (Figure 2). There were 14 patients with a low bleeding risk, of which 12 (85%) were prescribed thromboprophylaxis. Of the 54 patients with moderate to high bleeding risk, 24 (44%) were prescribed thromboprophylaxis. When comparing the proportion of thromboprophylaxis prescribing between patients with low versus moderate-high bleeding risk, there was a significant difference between the groups (*P* = .006); patients with moderate-high bleeding risk were less likely to be prescribed thromboprophylaxis (Table 3).

### Type of Thromboprophylaxis Prescribing and Associated Complications

Patients were 11.5 times more likely to receive LMWH (Dalteparin) than UFH, with 33 (92%), and 3 (8%) patients prescribed Dalteparin and UFH, respectively. For all gastroenterology patients, appropriate doses of UFH, and dalteparin were prescribed in accordance with VTE guidelines.^8^ A review of all re-admissions within 30 to 60 days did not show any VTE thromboprophylaxis complications. The changes in mean values of Hb and platelet levels from admission to discharge according to prescribed thromboprophylaxis is shown (Table 4). There was no significant difference in the mean Hb level change between patients that were prescribed thromboprophylaxis and those that were not (*P* = .164). There was also no significant difference in mean platelet level between patients that were prescribed thromboprophylaxis and those that were not (*P* = .749). Additionally, no patients experienced a platelet reduction greater than 50% or a Hb drop greater than 20 g/L (key thresholds that can be used as markers for potential medication related harm).

**Table 4.** Differences in Mean Values of Hemoglobin and Platelet in Gastroenterology Patients by Thromboprophylaxis Prescription (n = 68).

Hemoglobin change by prescribed thromboprophylaxis (n = 64)*			
Laboratory test	Prescribed thromboprophylaxis (n = 36)	Not prescribed thromboprophylaxis (n = 28)	Comparison between groups
	Mean Hb (g/L) (SD)	Mean Hb (g/L) (SD)	*P*-value
Hemoglobin change from baseline to discharge**	−7.17 (14.67)	−2.04 (14.25)	*P* = .164
Percentage platelet change by prescribed thromboprophylaxis (n = 62)*			
Laboratory test	Prescribed thromboprophylaxis (n = 35)	Not prescribed thromboprophylaxis (n = 27)	Comparison between groups
	Mean % (SD)	Mean % (SD)	
Platelet change (%) from baseline to discharge***	0.13 (27.32)	2.18 (22.76)	*P* = .749

* Four patients had missing hemoglobin data and six patients had missing platelet data.

** Hemoglobin is a measure for bleeding and a 20 g/L decrease was considered as clinically significant.

*** Platelets is a measure for HITT and 50% decrease was considered as clinically significant.

## Discussion

This study investigated the appropriateness of VTE prophylaxis regimens, and the quality and safety of thromboprophylaxis prescribing according to VTE and bleeding risk, for gastroenterology inpatients at a tertiary Australian hospital. All patients that had thromboprophylaxis prescribed had prescriptions which complied with the dosing, duration, and monitoring recommendations outlined in the Queensland Health State-wide VTE guidelines. However, completion and documentation of a VTE risk assessment was not always done. Thromboprophylaxis prescribing did not change significantly according to VTE risk assessed through Caprini scores, however thromboprophylaxis prescribing significantly decreased as bleeding risk according to HASBLED scores increased. Overall, there were no subsequent thromboprophylaxis-related complications from baseline to discharge.

Local VTE prophylaxis guidelines recommend that a VTE risk assessment is completed for all patients.^8^ However, in line with other studies, there was a lack of documentation for the completion of VTE risk assessments for hospital inpatients.^17^ Limited documentation can cause inefficiencies in workflow, with multiple clinicians completing the same assessment, which increases patients risk as appropriate thromboprophylaxis prescribing might be missed if it is assumed to have been completed already.^18^ With VTEs as a leading cause of preventable patient harm, this highlights the need to improve assessment, and documentation of VTE risk for hospital inpatients. The Australia and New Zealand Working Party on the Management and Prevention of Venous Thromboembolism have strategized that establishing electronic reminder systems may promote optimal use of thromboprophylaxis.^6^

One method to improve the screening and prescribing of thromboprophylaxis is using digital tools such as dashboards. Dashboards offer a novel approach for improving patient safety and optimizing high-risk therapies as digital dashboards can stream clinical data from electronic health records.^19,20^ Dashboards can also be coupled with a VTE stewardship program where a specialist pharmacist can work collaboratively, or as part of a team, to identify, explore, and escalate therapeutic problems to treating clinical teams for timely and corrective action.^21^ Pharmacist-led VTE stewardship has demonstrated reductions in mortality, LOS, bleeding complications, blood transfusion requirements, and cost of therapy.^22,23^ Such a dashboard has recently (March 2021) been implemented at this hospital. An audit of 1024 patients demonstrated that 73 (7%) patients had duplicate therapy (2 anticoagulants) prescribed, and on average 49 patients per day had dosing errors. This equates to 1470 errors each month and 17 885 errors each year. Future research will involve a robust study of the clinical and cost effectiveness of using this dashboard in practice as part of a VTE stewardship program.

### Limitations

This study was a retrospective review where the results are reliant on accurate, complete, and consistent documentation in patient clinical records. Undocumented VTE risk factors may result in calculations of lower Caprini scores which could underestimate the VTE risk for patients. Given that VTE risk assessments were often not documented, it was not possible to determine whether this was completed unless this was recorded in the patient’s clinical notes. The HASBLED scoring system was originally designed for patients with atrial fibrillation, therefore, if may not be applicable to this patient cohort. Due to the small sample size, we were unable to adequately assess the incidence of thromboprophylaxis-related complications of thrombotic and haemorrhagic events post discharge. Lastly, patients that presented to a private hospital would not be captured in this study. Despite these limitations, this study provides a basis for future research into the quality and safety of prescribed thromboprophylaxis for gastroenterology inpatients.

## Conclusion

The use VTE prophylaxis should be individualized in gastroenterology patients, with each patient assessed for benefits and potential risks, including VTE risk and bleeding risk. Adequate documentation of VTE risk assessment in this specific patient cohort is essential and needs to be improved for hospital inpatients.

## Supplemental Material

sj-docx-1-hpx-10.1177_00185787231172385 – Supplemental material for Evaluation of the Quality and Safety of Venous Thromboembolism Prophylaxis Among Gastroenterology Inpatients at a Tertiary Hospital in Australia Supplemental material, sj-docx-1-hpx-10.1177_00185787231172385 for Evaluation of the Quality and Safety of Venous Thromboembolism Prophylaxis Among Gastroenterology Inpatients at a Tertiary Hospital in Australia by Nameer van Oosterom, Keshia R. De Guzman, Karl Winckel, Anissa Johnson and Nazanin Falconer in Hospital Pharmacy

## Appendices

### Appendix A: Data Collection Variables for Each Patient

Variables collected for each patient:

1. Patient demographic details:
  - Age
  - Gender
  - Body mass index (weight and height)
2. Medical History of:
  - Inflammatory bowel disease
  - Smoking
  - Venous thromboembolisms
  - Haemorrhagic events
  - Stroke
  - Hypertension
  - Hepatic disease
  - Alcohol intake
3. Admission details:
  - Principle diagnosis/presenting complaint
  - Treating specialty team
  - Length of stay
  - If surgical intervention was required during admission, and if so, what surgery was completed
  - Occurrence of venous thromboembolism or haemorrhage during admission
  - Venous thromboembolism prophylaxis regimen used during admission
    - Drug
    - Dose
    - Frequency
    - Duration
    - If no prophylaxis is used, documented reason for no prophylaxis
  - Concurrent usage of antiplatelet drugs, and if so, which ones
  - Concurrent use of non-steroidal anti-inflammatory drugs
4. Laboratory function tests
  - Estimated glomerulus filtration rate (on admission and on discharge)
  - Platelet count (on admission and on discharge)
  - Haemoglobin (on admission and on discharge)
  - International normalised ratio
  - Creatinine
5. Readmission to hospital within 30 or 60 days, and if so, cause for readmission

### Appendix B:

**Table:** Modified Caprini and HASBLED Scoring system

Modified Caprini Score		
Risk factor	Criteria	Point
Age	≤40 y	+0
	41-60 y	+1
	61-74 y	+2
	≥75	+3
Surgery^a^	Yes	+1
	No	+0
Body mass index >25 kg/m^2^	Yes	+1
	No	+0
History of inflammatory bowel disease	Yes	+1
	No	+0
History of venous thromboembolism	Yes	+3
	No	+0
History/Active malignancy	Yes	+2
	No	+0
Length of stay^b^	<3 d	+0
	≥3 d	+2
Total score		*X* / 13

a Original Caprini score stratifies surgery as minor (+1), major (+2), or elective major lower extremity arthroplasty (+5). Major surgery is defined as length >45 minutes, laparoscopic (>45 minutes in length) or arthroscopic.

b Original Caprini score uses mobility rather than length of stay: normal mobility (+0), medical patient currently on bed rest (+1), patient confined to bed >72 hours (+2).

For feasibility of data collection, the modified Caprini score used any type of surgery as a dichotomous variable with surgery acquiring an additional 1 point. This is different to the original Caprini score which stratifies surgery into 3 categories, based on surgical techniques and length, each with different risk that may acquire up to 5 additional points. The original Caprini score also stratifies patient mobility into 3 categories that can acquire up to 2 additional points. Due to poor documentation of mobility status, length of stay was used as a surrogate marker for patient mobility in the modified Caprini score.^8^

**Table:**

HASBLED score		
Risk factor	Criteria	Point
Hypertension	Yes	+1
	No	+0
Renal disease	Yes	+1
	No	+0
Hepatic disease	Yes	+1
	No	+0
History of stroke	Yes	+1
	No	+0
History of major bleeding or predisposition to bleeding	Yes	+1
	No	+0
Elevated INR	Yes	+1
	No	+0
Age >65 y	<3 d	+0
	≥3 d	+1
Use of antiplatelets or NSAIDs	Yes	+1
	No	+0
Excessive alcohol intake	Yes	+1
	No	+0
Total score		X / 9

*Note*. Score calculated based on treating team admitting documentation of past medical history. Excessive alcohol intake was defined as ≥8 standard drinks per week, and an elevated INR was defined as >1.2. INR = international normalised ratio; NSAID = non-steroidal anti-inflammatory drug.
