# Supplementary material for: Evaluation of the Quality and Safety of Venous Thromboembolism Prophylaxis Among Gastroenterology Inpatients at a Tertiary Hospital in Australia
Source: Hosp Pharm. 2023 May 20;58(6):595–604. doi: 10.1177/00185787231172385 (PMC10977061; doi:10.1177/00185787231172385)
Supplement: sj-docx-1-hpx-10.1177_00185787231172385 – Supplemental material for Evaluation of the Quality and Safety of Venous Thromboembolism Prophylaxis Among Gastroenterology Inpatients at a Tertiary Hospital in Australia [file sj-docx-1-hpx-10.1177_00185787231172385.docx]

# Appendices

## Appendix A: Data collection variables for each patient

Variables collected for each patient:

1. Patient demographic details:
   1. Age
   2. Gender
   3. Body mass index (weight and height)
2. Medical History of:
   1. Inflammatory bowel disease
   2. Smoking
   3. Venous thromboembolisms
   4. Haemorrhagic events
   5. Stroke
   6. Hypertension
   7. Hepatic disease
   8. Alcohol intake
3. Admission details:
   1. Principle diagnosis / presenting complaint
   2. Treating specialty team
   3. Length of stay
   4. If surgical intervention was required during admission, and if so, what surgery was completed
   5. Occurrence of venous thromboembolism or haemorrhage during admission
   6. Venous thromboembolism prophylaxis regimen used during admission
      1. Drug
      2. Dose
      3. Frequency
      4. Duration
      5. If no prophylaxis is used, documented reason for no prophylaxis
   7. Concurrent usage of antiplatelet drugs, and if so, which ones
   8. Concurrent use of non-steroidal anti-inflammatory drugs
4. Laboratory function tests
   1. Estimated glomerulus filtration rate (on admission and on discharge)
   2. Platelet count (on admission and on discharge)
   3. Haemoglobin (on admission and on discharge)
   4. International normalised ratio
   5. Creatinine
5. Readmission to hospital within 30 or 60 days, and if so, cause for readmission

## Appendix B: Modified Caprini and HASBLED Scoring system

| Modified Caprini Score |  |  |
| --- | --- | --- |
| Risk factor | Criteria | Point |
| Age | ≤ 40 years | + 0 |
|  | 41 to 60 years | + 1 |
|  | 61 to 74 years | + 2 |
|  | ≥ 75 | + 3 |
| Surgery^1^ | Yes | + 1 |
|  | No | + 0 |
| Body mass index > 25 kg/m^2^ | Yes | + 1 |
|  | No | + 0 |
| History of inflammatory bowel disease | Yes | + 1 |
|  | No | + 0 |
| History of venous thromboembolism | Yes | + 3 |
|  | No | + 0 |
| History/Active malignancy | Yes | + 2 |
|  | No | + 0 |
| Length of Stay^2^ | < 3 days | + 0 |
|  | ≥ 3 days | + 2 |
| Total Score | | X / 13 |

^1^Original Caprini score stratifies surgery as minor (+ 1), major (+ 2), or elective major lower extremity arthroplasty (+ 5). Major surgery is defined as length > 45 minutes, laparoscopic (>45 minutes in length) or arthroscopic.

^2^Original Caprini score uses mobility rather than length of stay: normal mobility (+ 0), medical patient currently on bed rest (+ 1), patient confined to bed > 72 hours (+ 2).

For feasibility of data collection, the modified Caprini score used any type of surgery as a dichotomous variable with surgery acquiring an additional one point. This is different to the original Caprini score which stratifies surgery into three categories, based on surgical techniques and length, each with different risk that may acquire up to five additional points. The original Caprini score also stratifies patient mobility into three categories that can acquire up to two additional points. Due to poor documentation of mobility status, length of stay was used as a surrogate marker for patient mobility in the modified Caprini score.^8^

| HASBLED Score |  |  |
| --- | --- | --- |
| Risk factor | Criteria | Point |
| Hypertension | Yes | + 1 |
|  | No | + 0 |
| Renal Disease | Yes | + 1 |
|  | No | + 0 |
| Hepatic Disease | Yes | + 1 |
|  | No | + 0 |
| History of stroke | Yes | + 1 |
|  | No | + 0 |
| History of major bleeding or predisposition to bleeding | Yes | + 1 |
|  | No | + 0 |
| Elevated INR | Yes | + 1 |
|  | No | + 0 |
| Age >65 years | < 3 days | + 0 |
|  | ≥ 3 days | + 1 |
| Use of antiplatelets or NSAIDs | Yes | + 1 |
|  | No | + 0 |
| Excessive alcohol intake | Yes | + 1 |
|  | No | + 0 |
| Total Score | | X / 9 |

Score calculated based on treating team admitting documentation of past medical history. Excessive alcohol intake was defined as ≥8 standard drinks per week, and an elevated INR was defined as >1.2.

*INR* international normalised ratio, *NSAID* non-steroidal anti-inflammatory drug
